# The comparative effectiveness of decision aids in diverse populations with early stage prostate cancer: a study protocol for a cluster-randomized controlled trial in the NCI Community Oncology Research Program (NCORP), Alliance A191402CD

**DOI:** 10.1186/s12885-018-4672-3

**Published:** 2018-08-06

**Authors:** Joel E. Pacyna, Simon Kim, Kathleen Yost, Hillary Sedlacek, Daniel Petereit, Judith Kaur, Bruce Rapkin, Robert Grubb, Electra Paskett, George J. Chang, Jeff Sloan, Ethan Basch, Brittny Major, Paul Novotny, John Taylor, Jan Buckner, J. Kellogg Parsons, Michael Morris, Jon C. Tilburt

**Affiliations:** 1 Mayo Clinic, Rochester, MN USA; 2 University Hospitals, Case Western Reserve University, Cleveland, OH USA; 3 Regional Health, Rapid City, SD USA; 4 Mayo Clinic, Jacksonville, FL USA; 5 Albert Einstein Cancer Center, Bronx, NY USA; 6 Medical University of South Carolina, Charleston, SC USA; 7 Ohio State University, Columbus, OH USA; 8 MD Anderson Cancer Center, Houston, TX USA; 9 Alliance Statistics And Data Center, Mayo Clinic, Rochester, MN USA; 10 University of North Carolina, Chapel Hill, North Carolina, USA; 11 University of Chicago, Chicago, IL USA; 12 Moores UC San Diego Comprehensive Cancer Center, San Diego, CA USA; 13 Memorial Sloan Kettering Cancer Center, New York, NY USA

**Keywords:** Prostate cancer, Clinical trial, Decision aid, Shared decision-making

## Abstract

**Background:**

Treatments for localized prostate cancer present challenging tradeoffs in the face of uncertain treatment benefits. These options are best weighed in a process of shared decision-making with the patient’s healthcare team. Minority men experience disparities in prostate cancer outcomes, possibly due in part to a lack of optimal communication during treatment selection. Decision aids facilitate shared decision-making, improve knowledge of treatment options, may increase satisfaction with treatment choice, and likely facilitate long-term quality of life.

**Methods/design:**

This study will compare the effect of two evidence-based decision aids on patient knowledge and on quality of life measured one year after treatment, oversampling minority men. One decision aid will be administered prior to specialist consultation, preparing patients for a treatment discussion. The other decision aid will be administered within the consultation to facilitate transparent, preference-sensitive, and evidence-informed deliberations. The study will utilize a four-arm, block-randomized design to test whether each decision aid alone (Arms 1 and 2) or in combination (Arm 3) can improve patient knowledge and quality of life compared to usual care (Arm 4). The study, funded by the National Cancer Institute’s Community Oncology Research Program (NCORP), will be deployed within select institutions that have demonstrated capacity to recruit minority populations into urologic oncology trials.

**Discussion:**

Upon completion of the trial, we will have 1) tested the effectiveness of two evidence-based decision aids in enhancing patients’ knowledge of options for prostate cancer therapy and 2) estimated whether decision aids may improve patient quality of life one year after initial treatment choice.

**Trial registration:**

Clinicaltrials.gov: NCT03103321. The trial registration date (on ClinicalTrials.gov) was April 6, 2017.

## Introduction

Men with newly diagnosed localized prostate cancer face challenging treatment decisions. Surgery and radiation therapy are effective treatments, but each has different quality of life implications for men and their partners. These treatments, although potentially life-saving, impose their own burden related to treatment side effects. Some men may benefit from a monitoring approach called “active surveillance” if they have early, slow-growing prostate cancer. Making the right treatment choice depends of men being given all appropriate options and making sure they have a high-quality conversation with their specialist. This process creates substantial cognitive and emotional burden. Identifying a course of treatment that accords with patient goals and preferences for cancer control while attending to important quality of life trade-offs is crucial to minimizing the overall burden of the prostate cancer. Thus, prostate cancer treatment provides a crucial opportunity for patients and clinicians to engage in shared decision-making.

Prostate cancer disproportionately affects African-American (AA) men. Previous studies suggest that AA men have a higher incidence of more aggressive or advanced stage prostate cancer and cancer-specific mortality compared to the general population. [1–4] American Indian men from the Northern and Southern Plains also experience disparities in prostate cancer stage and survival comparable to AA men; prostate cancer is also the second leading cause of cancer-related mortality in American Indian men overall. [5, 6] Historically, AA men are less likely to receive radiation therapy or undergo surgery, and more likely to receive “watchful waiting” or active surveillance, despite having a higher incidence of intermediate and high-risk prostate cancer. [7–13] Minority men who undergo definitive therapy are more likely to experience treatment regret and greater functional outcome burden. [14, 15] Although little research has been dedicated to treatment variation in American Indian men, a recent report suggested that this underserved population also has lower rates of definitive treatment following a diagnosis of prostate cancer. [5]

American Indian and Alaska Native (AI/AN) men experience greater prostate cancer mortality than non-Hispanic whites. [6] In Hispanic/Latino men disparities are less clear. In general, although national data do not suggest major outcome disparities in this group, local and regional studies and patterns-of-care studies review pockets of disparities particularly related to delays in care or different treatment patterns for Hispanic/Latino men. [16–22]

Minority men in general experience disparities in prostate cancer knowledge and care patterns, and they suffer from more functional outcome morbidity in prostate cancer. [1–4] Combined, these disparities compound known disease burden differences in these populations. Studies have documented lower levels of knowledge about prostate cancer among AA men compared with other racial/ethnic groups. [23] Poorer outcomes among minority prostate cancer patients may arise from factors beyond healthcare access. Worse functional outcomes result from overly aggressive treatment, while worse mortality outcomes likely result from under-aggressive treatment. On the one hand greater use of aggressive therapies could save lives, but could at the same time exacerbate existing disparities in functional outcomes associated with aggressive therapy.

Disparities also may be rooted, at least in part, in preference-discordant treatment choices stemming from poor communication between physicians and their minority patients. Shared decision-making (SDM) may be especially difficult to achieve when patients’ literacy and culturally mediated values challenge the biomedical establishment’s attempts to communicate the complexity surrounding modern treatment choices. In addition to myriad patient and health system factors including no/under insurance, ability to get insurance coverage, access to health care services, access to second opinions, and the influence of common comorbid health conditions, communication breakdown (failure to achieve SDM) may compound racial/ethnic disparities in treatment outcomes.

Decision aids have been shown to improve shared decision-making in a growing variety of clinical decisions. [24] Decision aids vary from information-centric tools designed to help patients self-educate about benefits and burdens of treatment choices, to more visually oriented “conversation pieces” that foster and facilitate preference-sensitive conversation between patient and physician. [25] Shared decision-making tools may enable deliberation about treatment choices in contexts where cultural differences and social determinants of health complicate fully ascertaining patient preferences. Thus, meaningful progress in addressing racial disparities in prostate cancer treatment may be possible by facilitating shared decision-making through the use of decision aids.

Because choosing the right treatment in prostate cancer is so challenging, it requires high quality conversations. Because communication breakdowns may be to blame for documented disparities in the provision of prostate cancer treatments to minorities—particularly African American and American Indian men—we designed this study to test known methods of improving conversations between clinicians and patients in a trial that seeks to preferentially enroll minority men confronted with a new diagnosis of prostate cancer. The overall goal of our trial is to test the comparative effectiveness of two decision aids—an information-rich decision aid tool (*Knowing Your Options*) delivered before specialist consultation and a conversation-facilitating decision aid tool with fewer details (*Prostate Choice*) delivered during specialist consultation—in a four-arm trial testing each decision aid alone and in combination compared to usual care. Patient knowledge about prostate cancer treatments will be our primary outcome measured immediately after the index consultation with a urology specialist. We will oversample minority populations to determine whether the decision aids mitigate disparities across race/ethnic groups in their measured knowledge of prostate cancer and its treatments.

## Methods

### Design

We will use a cluster-randomized controlled trial to compare the effectiveness of the two decision aids alone and in combination. The trial will feature four arms. Two arms will incorporate one of the two decision aids. A third arm will incorporate both decision aids, and the fourth arm will be usual care (i.e., no intervention). Randomization will occur on the site level—entire urology practices will be randomized to one of the study arms. This will protect against contamination, a major concern for studies comparing care delivery interventions. [26] The study will be conducted in clinical settings where patients have recently learned about their diagnosis of localized prostate cancer and are receiving their first consultation about treatment options.

### Setting

Our study will be conducted among institutions which are components of National Cancer Institute’s National Community Oncology Research Program (NCORP) sites. While the parent NCORP Research Base for this trial is the Alliance for Clinical Trials in Oncology (Alliance), members of other bases (SWOG and ECOG-ACRIN) will also be allowed to participate. These groups are members of the National Cancer Institute (NCI) National Clinical Trials Network (NCTN). The trial is sponsored by the Alliance Disparities and Cancer Care Delivery Research (CCDR) committees and funding for the study is available to NCTN group members as part of NCORP CCDR-designated award funds. NCI defines CCDR research as “multidisciplinary science that examines how patient and clinician behavior, organizational structures, health technologies, and financing approaches influence the availability, quality, cost, and outcomes of cancer care. CCDR generates evidence that can be used to improve clinical practice patterns as well as develop and test promising interventions within the health care delivery system.” [27] Several participating sites are designated by NCORP as “Minority / Underserved” research centers and have demonstrated success in reaching our target minority populations.

### Participants

#### Inclusion and exclusion criteria

Our trial will enroll men with a new diagnosis of non-metastatic prostate cancer. Eligible participants must have a prostate biopsy not older than 4 months at the time of enrollment. Patients may have a Gleason score from 6 to 10 and must have a prostate-specific antigen (PSA) less than 50 ng/ml. Patients must be enrolled in the study after notification of a positive biopsy but before receiving any consultation about treatment options. Patients presenting for a second opinion are not eligible. Patients must be able to read and comprehend English. In lieu of this requirement, an English-proficient caregiver or clinical / research support staff may assist participants in reading or translation analogous to clinical care. Participants will not be enrolled who have had another non-cutaneous malignancy in the previous 5 years. Patients with a history of non-melanoma skin cancer are eligible to participate. Our trial will oversample African American (AA), American Indian/Alaska Native (AI/AN), and Hispanic (HS) men. At least 50% of the study’s total enrollment will draw from these target populations. Sites will be instructed to limit recruitment of men from other racial and ethnic subgroups to 50% to achieve minority recruitment targets.

#### Site recruitment

**Fig. 1:**
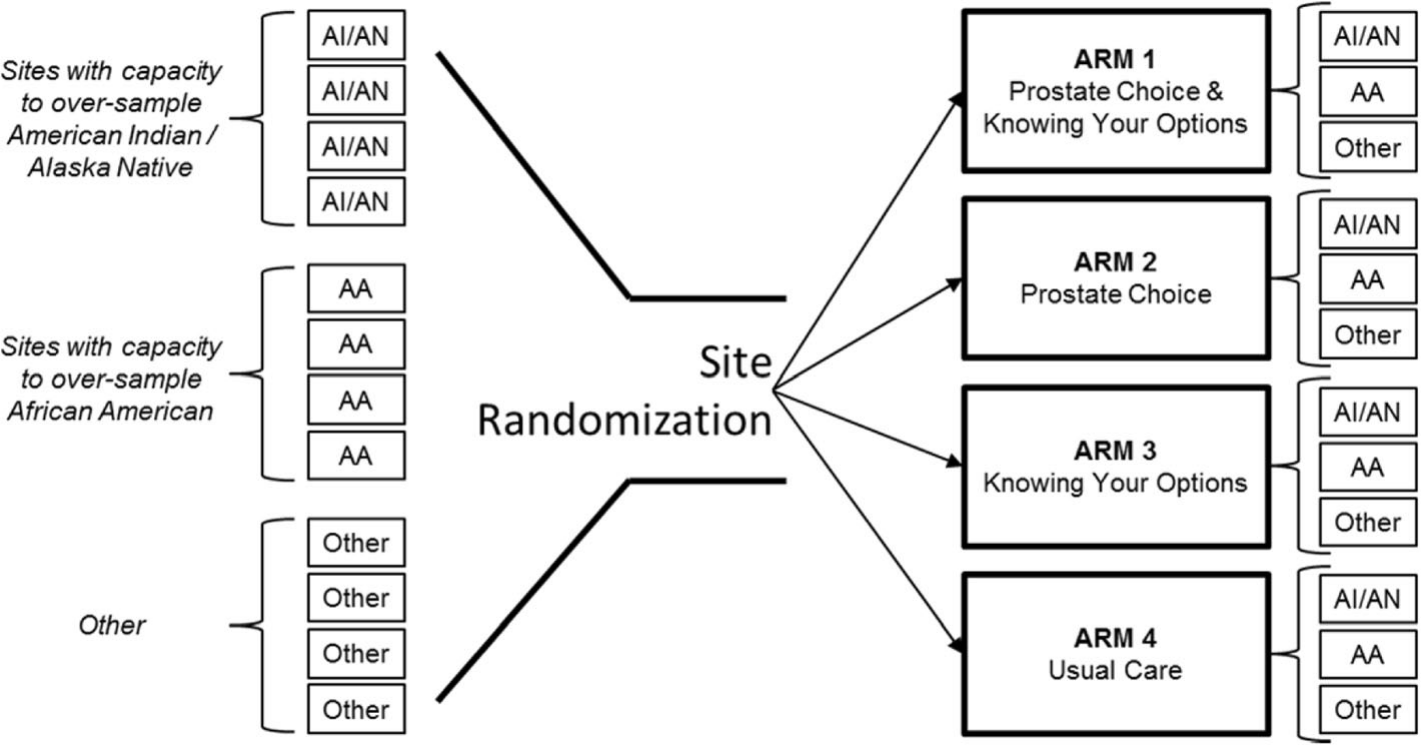
Site Randomization

Because of the study design and target enrollment goals, a sufficient number of minority-oriented sites have been identified. The block-randomization study design includes 20 participating sites divided equally across the four arms (see Fig. 1), and each site must recruit similar numbers of participants based on our power calculations. In order to participate in our trial, urology practices must be rostered as funded components of the NCORP institutions who receive CCDR funds. In addition to the requirements of the funding structure, qualifying sites must also have urology practices with urologists who are capable and willing to deliver decision-aid interventions in conjunction with their standard care practices for patients with new prostate cancer diagnoses. These requirements for site eligibility require significant communication between the study team and sites meeting the NCORP criteria, to determine which sites have the capacity and interest to participate.

#### Participant recruitment

Participant recruitment will remain flexible to accommodate each site’s workflow for notifying patients about new cancer diagnoses and providing consultation about treatment choices. Some sites disclose positive cancer diagnoses by phone, with the treatment consultation occurring days later. Other sites combine notification and treatment discussion into a single consultation with the physician provider. In all cases, participating sites will need to ensure that registration and intervention (in applicable study arms) occur after diagnosis notification and prior to the specialist consultation. Each site will develop methods for identifying eligible patients ahead of visits and for recruiting patients in a way that avoids the possibility of inadvertent diagnosis disclosure by study staff.

### Interventions

The trial intervention arms consist of one or both decision aids targeting men with non-metastatic prostate cancer. The decision aids are designed to convey information about prostate cancer and its treatments in order to enable patients to make more informed treatment choices under the guidance of their physician. Neither decision aid is intended to displace fundamental aspects of the consultation or constrain physicians’ ability to make treatment recommendations. Two decision aids are being tested in our trial—the *Prostate Choice* tool which was developed and tested by the study team, and the *Knowing Your Options* tool developed by the Agency for Healthcare Research and Quality. [28]

#### Prostate choice

The *Prostate Choice* decision aid was originally developed by members of the study team in 2011. In preparation for the trial reported here, the decision aid was revised, and culturally and cognitively tested in focus groups comprising members of our target minority populations. It is a “text-light” tool incorporating the best available evidence in a literacy-sensitive, web-based design to orient patients toward the range of considerations and goals for prostate cancer therapy, including cancer control and quality of life implications. The tool incorporates clinical variables including patients’ age, PSA, primary and secondary Gleason scores, clinical staging, and number of positive and negative biopsy cores. These data are used to return a D’Amico risk category [29] in a summary screen in the tool. The tool also collects co-morbidity variables to return an age and co-morbidity adjusted life expectancy on the summary screen. Patient quality of life priorities are also gathered via the EPIC 26 prostate cancer quality of life measure [30] and some simple visual analogue scales eliciting patients’ relative priorities regarding cancer control, bowel and urinary control, and sexual function. All results are provided on a single summary screen along with options for viewing summary information about treatment modalities in pop-up screens for in-visit use. Importantly, active surveillance is presented as a peer-level “therapy” along with surgical and radiation options and hormone therapy. The summary screen becomes the main focus of attention in the consultation, and it allows patients to “drive” the conversation by gravitating toward the elements on the summary screen that are most salient to their decision-making intuitions. The *Prostate Choice* tool is not intended to constrain clinician advice regarding treatment choices. The specialty clinician may incorporate the tool and still make clinical recommendations, including strongly encouraging or discouraging certain treatment options. The goal, however, is to situate those recommendations within patient-driven, preference-sensitive education in the range of treatments and their situation-specific strengths and limitations. Participating clinicians will be given brief orientation videos to explain the tool’s use and on-site training by study staff is available on an as-needed basis.

#### Knowing your options

The *Knowing Your Options* tool is a publicly available web-based tool designed and supported by the Agency for Healthcare Quality and Research (AHRQ): https://effectivehealthcare.ahrq.gov/topics/decision-aids/prostate-cancer. In contrast to the *Prostate Choice Tool*, the *Knowing Your Options Tool* (KYO) is text-heavy, with multiple screens and requiring significant page scrolling. Prostate cancer and specific information about its diagnosis and prognosis are described in detail, and the range of treatment options are described and visualized. The *Knowing Your Options* tool is an evidence-based tool that was originally designed to be used by the patient outside of and prior to a specialist consultation (perhaps at home) to enhance the treatment decision-making process. Similar to *Prostate Choice*, KYO also collects users’ relevant clinical information for prostate cancer severity and risk of cancer-specific mortality. KYO also queries patients about quality of life priorities relevant to the different primary treatment options for prostate cancer. To our knowledge the efficacy of KYO in increasing patients’ knowledge about prostate cancer treatment options has not been formally tested.

### Outcomes and data collection

#### Primary outcome: Knowledge

**Fig. 2:**
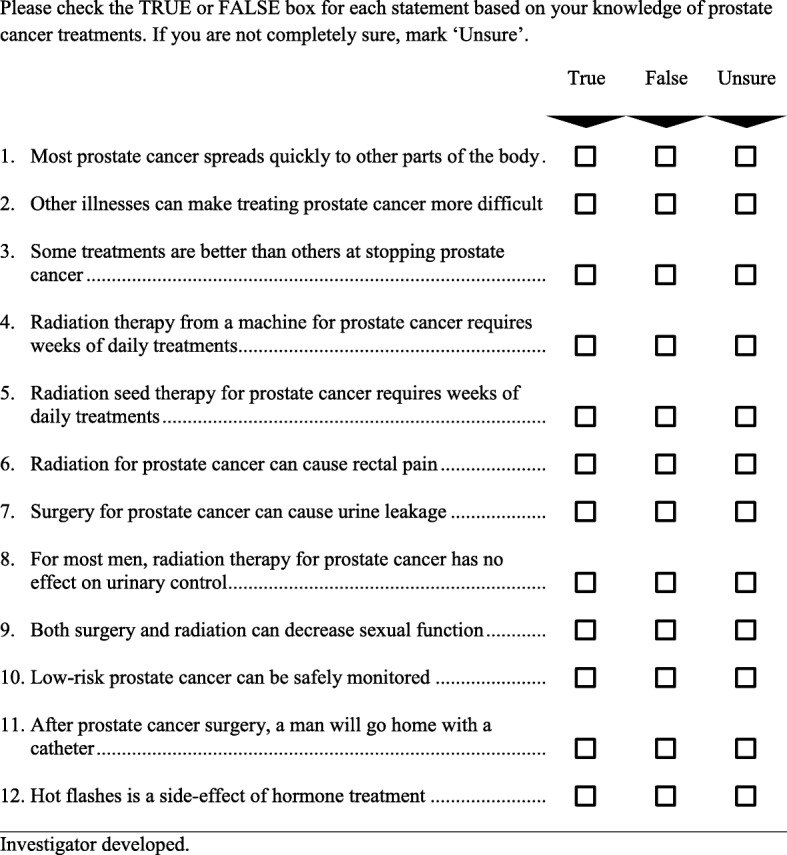
Prostate Cancer Treatments Questionnaire

The primary outcome of our study is knowledge about prostate cancer treatments measured immediately after the consultation with the urologist. While consensus is lacking on how to measure shared decision-making, measuring knowledge about treatment options is commonly used as a reliable proxy. [31] To measure knowledge, we designed a 12-item knowledge measure—the Prostate Cancer Treatment Questionnaire. The items for this measure were identified by urology experts based on content validity of clinical consideration of essential knowledge needs for patients facing decisions about prostate cancer treatment (see Fig. 2). As a pragmatic measure, our instrument omits items about prostate cancer anatomy and physiology and focuses instead on questions regarding disease severity and the implications of the major treatment modalities for survival and quality of life. We conducted cognitive testing of draft measures with 10 prostate cancer survivors to ensure that respondents understand the questions as intended, that the questions are interpreted consistently by all respondents, and that respondents are willing to answer the questions. [32] Cognitive testing and input from our patient advocate advisory panel led to refinement of the items. Institutional review board (IRB)-approved pilot testing was then conducted in 45 men presenting at the urology department at Mayo Clinic for consultation about treatment choices for a new diagnosis of prostate cancer. Preliminary analyses confirmed that the measure targeted a moderate level of knowledge and could be used to identify improvement in knowledge (i.e., the measure did not suffer from ceiling effects). The 12 items were moderately correlated (Cronbach’s alpha of 0.62). Knowledge scores (number of correct answers) were significantly correlated in a hypothesized direction with higher educational attainment (*p* = 0.02), evidence of concurrent validity. (i.e. knowledge scores increase with increasing levels of educational attainment).

#### Secondary outcomes: Decisional conflict and regret

The decisional conflict scale was developed and validated by O’Connor [33] as an instrument intended to “elicit 1) health-care consumers’ uncertainty in making a health-related decision; 2) the factors contributing to the uncertainty; and 3) health-care consumers’ perceived effective decision-making.” The low literacy version of this questionnaire will be used, and it contains 10 items answered on a 3 point scale (i.e., “yes,” “unsure,” “no”) and may be adapted to specific health-care decision scenarios. Example questions include agreement with the following statements: “Did you know which options were available to you?”, “Did you know the benefit of each option?,” “Did you feel sure about what to choose?” The questionnaire will be administered once, immediately after the consultation (post-consultation). We estimate it will take participants approximately 5–7 min to complete this questionnaire. O’Connor has identified meaningful decisional conflict thresholds—scores less than 25 (associated with implementing decisions) and scores above 37.5 (associated with decision delay and uncertainty). [34] Decisional conflict will serve as an important corroborating measure in our assessment of effectiveness of decision aids. At 12 months, we will administer the Decision Regret Scale (also designed and validated by O’Connor and colleagues). [35] Decisional Regret has been correlated with Decisional Conflict. The Decisional Regret scale is a short, 5-item scale measuring “distress or remorse after a (health care) decision.” Questions are answered on a 5-point agreement scale.

#### Exploratory secondary outcomes: Treatment choice and quality of life

At 12 months post-intervention we will measure patient’s quality of life via the Expanded Prostate Cancer Index Composite (EPIC-26) quality of life scale for urologic functioning. This instrument measures health-related quality of life and returns summary scores for urinary, bowel, sexual, and hormonal domains with high test-retest reliability and internal consistency. As an exploratory aim, we will analyze 12-month quality of life (QOL) data to check for minority subgroup difference and differences between intervention arms. At 12 months we will also ascertain via chart review the patient’s treatment choice following the intervention. Treatment utilization will be categorized by the type of treatment the patient had (surgery vs. radiation vs. active surveillance). If we accrue > 10% of our study population among those whose primary language is Spanish, we will conduct exploratory analyses on differential effects of the intervention in this sub-group.

### Statistical considerations

#### Sample size

A recent Cochrane review suggests that most patients can accurately answer 50% (standard deviation of 12%) of the questions asked of them. [36] On average, decision aids (DAs) increase that knowledge by at least a 20% (and in some cases as high as 60%) increase in questions asked being answered correctly, but 95% of trials show absolute knowledge increases of 10% or greater. We will consider an absolute 8% or larger increase (equivalent to one additional item answered correctly in our 12-item measure) in knowledge as clinically meaningful for either the during-consultation or pre-consultation DA in this clinical trial. The four arms of this study make up a 2 X 2 factorial design. Thus, it is natural to consider evaluating the decision aids using a two-way analysis of variance (ANOVA). The two factors in the ANOVA will be 1) having received during-consultation Prostate Choice (yes or no) and 2) having received pre-consultation DA (yes or no). We will consider simultaneously testing (at a significance level of 0.025 for each test) the main effects of the two decision aids as our primary analysis. That is, we will simultaneously test the null hypothesis that the average knowledge (i.e., the percent of correct responses to questions) among those who received the pre-consultation DA is equal to that among those who did not (vs. an alternative that these two averages are not equal), and the null hypothesis that the average knowledge among those who received the during-consultation *Prostate Choice* is equal to that among those who did not (vs. an alternative that these two averages are not equal).

A sample of 100 patients (25 patients per arm) would give us approximately 85% power to detect a difference between those receiving pre-consultation DA and those not receiving pre-consultation DA, under the alternative that the average knowledge among those receiving pre-consultation DA is 58%, and that the average knowledge among those not receiving pre-consultation DA is 50%, using a two-sample t-test (with two-sided alternative) with a 2.5% significance level (this is equivalent to the F test for the main effects in the ANOVA). Under a similar alternative, the same can be said for the during-consultation *Prostate Choice* decision aid. Thus, if patients within each site were not correlated with each other, our target sample size would be 100 patients. There will be some, but insufficient power to detect an interaction between the two decision aids, but such effects are not anticipated in this study. Therefore, we will not test for such an interaction in the primary analysis. Since we expect *k*=20 sites to participate in this clinical trial, we would need about *m* = 5 patients to be enrolled from each site (on average) to achieve a total enrollment of 100 patients.

We cannot assume that participants within each site will be independent of each other given our design. Our actual sample size estimate accounts for clustering by site. Assuming the intra-site correlation coefficient ρ will be approximately 0.1 (rather than zero) for all study sites, we inflate the target sample size by a factor [37] of 1 + (m-1)ρ = 1 + (5–1)*0.1 = 1.4 to achieve comparable power to that in a patient-level randomized trial. We would then target an effective sample size of 140 patients (approximately 35 patients per arm, 7 patients per site). To account for withdrawal and loss to follow-up for longer term secondary outcomes and allow increased power to detect racial/ethnic differences, we have further inflated the total sample size by 20% to a total number of 172 patients. These 172 patients, recruited from 20 participating sites (about 8–9 patients per site) will receive the intervention (or control) to which their location is randomized. Of these, we anticipate recruiting 86 men from African American, American Indian race, and/or Hispanic/Latino ethnicity.

#### Analysis of primary outcome

The primary outcome, knowledge, will be assessed by a standardized questionnaire (the Prostate Cancer Treatment Questionnaire) administered once, immediately after the clinical consultation while the patient is still at the study site. The number correct from this 12-item measure will be scored as a percent.

A pre-post method for measuring knowledge was considered. However, several factors led us to favor a one-time post-intervention measurement: 1) Our study’s randomized design should control for differences in baseline knowledge; 2) a pre-post design could be confounded by learning effects associated with the baseline measurement since the baseline and post-intervention measurements would only be 1–2 h apart. Such learning affects could lead to artificial improvements in our control group which could limit our ability to see “true” differences attributable to the intervention(s); and 3) a one-time measurement of knowledge will minimize burden to respondents, particularly during the consenting and baseline measurement period where we attempt to impose as little disruption to clinical workflows as possible.

Although the randomization unit will be the participating site, our inferential unit for statistical analysis will be the individual patient. Due to the potential for correlation among patients within the same site, a mixed effects regression model (also known as random effects model or multi-level model) will be utilized to examine the effects of the during-consultation *Prostate Choice* and the pre-consultation *Knowing Your Options* decision aids. [38] Specifically, this model will contain a fixed intercept, a fixed effect for having received *Prostate Choice*, a fixed effect for having received *Knowing Your Options*, and a random, site-specific intercept to allow patients within the same site to be correlated. Baseline patient-level characteristics including race, ethnicity, cancer stage and grade, and site-level characteristics may be incorporated in this model if deemed appropriate. A similar approach will be utilized in the statistical analysis of secondary endpoints. Furthermore, descriptive statistics will be reported after incorporating cluster information, particularly the empirical cluster size, and the observed intra-cluster correlation.

An interim analysis will be used to test if the intervention arm (either during-consultation *Prostate Choice* or *Knowing Your Options* pre-consultation DA) has produced better knowledge than the respective control arm. This study will also be monitored for futility. At interim analysis, a 95% one-sided confidence interval on the difference of knowledge between the intervention and control arm will be computed. If the confidence interval does not cover the target alternative of 0.1 for one of these comparisons, the DSMB may consider stopping the trial early.

#### Analysis of secondary outcomes

Decisional quality, average clinical time required, and patient QOL scores will be compared across study arms using linear mixed models similar to that used to assess the primary endpoint. In particular, this model will include fixed effects for *Prostate Choice* and *Knowing Your Options* and a random, site-specific intercept to allow for subjects within the same site to be correlated. Utilization will be compared across DA types using a generalized linear mixed model, again with fixed effects for having received *Prostate Choice* and having received *Knowing Your Options* and a random, site-specific intercept.

As an additional secondary objective, we will explore whether the overall effects of interventions on patient knowledge, quality of life, and treatment utilization differ by racial/ethnic subgroups. Our sample size is driven by the primary outcome of knowledge. Oversampling of minority populations of interest will achieve a robust representation of these minority populations in our final sample, but we have not designed the trial to have sufficient power to ascertain subtle subgroup differences in knowledge and quality of life by race/ethnicity subgroups. These secondary analyses will be exploratory, because fully testing the racial/ethnic differences would require prohibitively large sample sizes, and the literature does not suggest a strong race-based rationale for differences. We anticipate enrolling approximately 50% minority men of our overall sample (*n* = 86). This sample would give us approximately 78% power to detect an absolute difference of 8% in knowledge for either of the decision aids’ main effects using a two-sample t-test (with two-sided alternative) with a 2.5% significance level (i.e. the same analysis/assumptions used to power the primary analysis). If subtle but potentially important trends in subgroup differences are identified in these exploratory analyses, those findings could be used to justify a larger study examining a primary hypothesis related to racial/ethnic difference or could influence the design of subsequent culturally tailored interventions. At present, the science of decision aids and the state of the evidence surrounding racial/ethnic differences in the effect of decision aids would not support testing such a hypothesis as a primary endpoint.

## Discussion

Preference-sensitive decisions involve uncertainty about net outcome benefit, making patient values and preferences paramount in the treatment decision. [39–41] Because of the lack of clinical trial data suggesting a superior initial active prostate cancer therapy, physicians should help their patients successfully deliberate about the quality of life implications and burdens of different primary treatments to reach a decision that embodies the principles of shared decision-making (SDM). SDM is a model of evidence disclosure and values elicitation intended for preference-sensitive decisions and is endorsed by all major professional societies. [42–45]

By incorporating decision aids into the patient experience of receiving clinical guidance and treatment for prostate cancer, we may make critical progress toward shared decision-making in urologic oncology, especially in those patients whose cultural affinities add complexity to effective communication between provider and patient. Decision aids that are sensitive to cultural norms and that enable patient-driven conversation about treatment options for prostate cancer may hold one of the keys to reducing known disparities in prostate cancer treatment and outcomes. At the conclusion of our trial, we will have data showing the impact of decisions aids on patient knowledge in a sample enriched with minority men with new diagnoses of prostate cancer.
